# Ethylene Signaling Is Required for Fully Functional Tension Wood in Hybrid Aspen

**DOI:** 10.3389/fpls.2019.01101

**Published:** 2019-09-26

**Authors:** Carolin Seyfferth, Bernard A. Wessels, András Gorzsás, Jonathan W. Love, Markus Rüggeberg, Nicolas Delhomme, Thomas Vain, Kamil Antos, Hannele Tuominen, Björn Sundberg, Judith Felten

**Affiliations:** ^1^Umeå Plant Science Centre, Department of Plant Physiology, Umeå University, Umeå, Sweden; ^2^Department of Chemistry, Umeå University, Umeå, Sweden; ^3^Arevo AB, Umeå, Sweden; ^4^Institute for Building Materials, Swiss Federal Institute of Technology Zurich (ETH Zurich), Zurich, Switzerland; ^5^Laboratory of Wood Materials, Swiss Federal Laboratories of Materials Science and Technology, Dubendorf, Switzerland; ^6^Umeå Plant Science Centre, Department of Forest Genetics and Plant Physiology, Swedish University of Agricultural Sciences, Umeå, Sweden; ^7^DIADE, Univ Montpellier, IRD, Montpellier, France; ^8^Department of Integrative Medical Biology, Umeå University, Umeå, Sweden; ^9^Stora Enso AB, Nacka, Sweden

**Keywords:** xylem, wood, ethylene, tension wood, lignin, microfibril angle, Raman microspectroscopy, transcriptomics

## Abstract

Tension wood (TW) in hybrid aspen trees forms on the upper side of displaced stems to generate a strain that leads to uplifting of the stem. TW is characterized by increased cambial growth, reduced vessel frequency and diameter, and the presence of gelatinous, cellulose-rich (G-)fibers with its microfibrils oriented parallel to the fiber cell axis. Knowledge remains limited about the molecular regulators required for the development of this special xylem tissue with its characteristic morphological, anatomical, and chemical features. In this study, we use transgenic, ethylene-insensitive (ETI) hybrid aspen trees together with time-lapse imaging to show that functional ethylene signaling is required for full uplifting of inclined stems. X-ray diffraction and Raman microspectroscopy of TW in ETI trees indicate that, although G-fibers form, the cellulose microfibril angle in the G-fiber S-layer is decreased, and the chemical composition of S- and G-layers is altered than in wild-type TW. The characteristic asymmetric growth and reduction of vessel density is suppressed during TW formation in ETI trees. A genome-wide transcriptome profiling reveals ethylene-dependent genes in TW, related to cell division, cell wall composition, vessel differentiation, microtubule orientation, and hormone crosstalk. Our results demonstrate that ethylene regulates transcriptional responses related to the amount of G-fiber formation and their properties (chemistry and cellulose microfibril angle) during TW formation. The quantitative and qualitative changes in G-fibers are likely to contribute to uplifting of stems that are displaced from their original position.

## Introduction

When angiosperm tree stems are displaced from their original growing position, due to external factors including wind, snow load, and/or growth on uneven terrain, they form tension wood (TW) in order to reorient/uplift the stem towards its original growth position (Felten and Sundberg, 2013). TW formation is characterized by asymmetric xylem growth in the stem, originating from enhanced activity of the secondary cambium at the upper (TW) side of the stem as compared with the lower [opposite wood (OW)] side (Timell, 1986). TW is characterized by an increased fiber-to-vessel ratio compared with OW (Esau, 1977). Another typical feature of TW is the presence of an altered cell wall layer structure in fiber cells. In many tree species, TW fibers [called gelatinous (G)-fibers] are characterized by the presence of a tertiary cell wall layer, mainly composed of a gelatinous, cellulose-rich layer (G-layer) with microfibrils having high crystallinity (Müller et al., 2006). Typically, a bimodal distribution of the cellulose microfibril angle (MFA) is observed in TW fibers using X-ray diffraction. The S2-layer exhibits an MFA of 20–40°, which is considerably larger than the values reported for the S2 of normal wood (NW) (5–15°), whereas the G-layer shows a very small MFA of 0–5° (Müller et al., 2006; Goswami et al., 2008; Rüggeberg et al., 2013). The highly porous structure of the G-layer (Norberg and Meier, 1966; Gierlinger and Schwanninger, 2006; Clair et al., 2008; Chang et al., 2009) allows for water incorporation resulting in a gelatinous aspect, from which the name gelatinous- or G-layer originates. The G-layer with its low MFA together with the S2 layer with its high MFA is thought of as a crucial factor to establish the tensile force that leads to the uplifting of the tree in response to the initial displacement (Goswami et al., 2008; Mellerowicz and Gorshkova, 2012). Even though the G-layer formation is a common phenomenon observed in the TW of many tree species, numerous tree species form TW without typical G-fibers, but rather with other cell wall layer modifications that all have small MFAs in common, which similarly exerts a tensile strain that leads to re-orientation of the stem (summarized in Ruelle, 2014).

In aspen, the plant hormone ethylene (ET) has been demonstrated to influence many of the features that characterize TW formation (Love et al., 2009; Felten et al., 2018). Indeed, after stem displacement, ET biosynthesis increases on the TW side in parallel with an asymmetric induction of 1-aminocyclopropane-1-carboxylate oxidase (ACO), which converts the ET precursor 1-aminocyclopropane-1-carboxylic acid (ACC) to ET (Andersson-Gunnerås et al., 2003). Furthermore, exogenous application of ET or ACC could mimic all typical TW characteristics such as enhanced cambial growth, increased fiber-to-vessel ratio and induction of the G-layer formation (Felten et al., 2018). Finally, the role of endogenous ET signaling in the unilateral growth response induced by leaning was investigated by constructing ET-insensitive (ETI) hybrid aspen trees. This study demonstrated an overall reduction in the TW/OW ratio in the ETI trees compared with wild-type trees (Love et al., 2009). Whether this reduced asymmetry affected the functionality of the TW was, however, not shown. Also, whether endogenous ET induced by leaning mediates the secondary wall modification in fibers observed in TW remains to be evaluated.

TW-forming tissues exhibit a strongly modified transcriptome (Paux et al., 2005; Andersson-Gunnerås et al., 2006; Chen et al., 2015; Zinkgraf et al., 2018), and it seems plausible that this is at least partly regulated through ET signaling. ET is perceived by the ET receptors localized at the endoplasmic reticulum (Bleecker et al., 1988; Cancel and Larsen, 2002). Ethylene binding derepresses the downstream ET signaling cascade (Merchante et al., 2013; Xu and Zhang, 2015). This leads to phosphorylation of the downstream component ETHYLENE-INSENSITIVE 2 (EIN2) and the cleavage of the EIN2 C-terminus (EIN2Cend) that translocates into the nucleus. The translocation triggers the expression of the ETHYLENE-INSENSITIVE 3 (EIN3)/EIN3-LIKE 1 (EIL1) transcription factors (TFs) and stabilizes EIN3/EIL1 proteins by preventing their proteasomal degradation (Qiao et al., 2009; Wen et al., 2012; Li et al., 2015; Merchante et al., 2015; Zhang et al., 2016). It has long been thought that EIN3/EIL1 activates the expression of ETHYLENE RESPONSE FACTORS (ERFs), which then trigger expression of their target genes through binding to GCC-boxes in the promoter region (Solano et al., 1998; Zhang et al., 2011; Shi et al., 2012). Recently, however, EIN3/EIL1 has been shown to directly bind to the promoter of many targets (beyond ERFs) and to modulate their expression (Chang et al., 2013). Furthermore, the ET downstream genes that were activated in hybrid aspen by exogenously applied ACC more frequently contained the EIN3/EIL1-binding motif (TEIL motif) in their promoter rather than the GCC-box (ERF-binding motif), and only a few ACC-activated ERFs contained the TEIL motif in their promoter (Felten et al., 2018). Therefore, it is likely that both ERFs and EIN3/EIL1 connect the ET signaling pathway to the molecular program of (tension) wood formation. In NW, we have identified *in silico* that ERFs and EIN3/EIL1 members act as hubs for regulating genes involved in wood formation (Seyfferth et al., 2018). Also, certain ERFs are transcriptionally induced in TW (Vahala et al., 2013).

We hypothesize here i) that ET signaling is required for all aspects of TW formation (cambial activity, vessel differentiation, G-layer formation, and MFA orientation) and that TW in ETI trees lacks these typical TW features and consequently the capacity to upright an inclined stem, and ii) that there is a molecular connection on transcriptional level between ET signaling and the molecular pathways regulating the typical developmental modifications in TW. To test these hypotheses, we conducted leaning experiments with wild-type and ETI hybrid aspen (*Populus tremula* L. × *Populus tremuloides* Michx) trees (Love et al., 2009), and we investigated how ET insensitivity affected TW formation, the uplifting response, and transcriptome changes in TW-forming tissues.

## Materials and Methods

### Biological Material and Growth Conditions

The genetic background of the trees used in this study was hybrid aspen (*P. tremula* L. × *P. tremuloides* Michx.) clone T89. The strongest ETI genotype, according to previous experiments (Love et al., 2009; Felten et al., 2018), *pLMX5::etr1-1*, was chosen (line 6E) for all experiments. Transgenic and wild-type trees were transferred to soil after 4 weeks of *in vitro* propagation (Nilsson et al., 1992). For TW experiments (note: conditions specific for uplifting experiments are different and stated below), trees were propagated *in vitro* (for details see Felten et al., 2018) and at about 8- to 10-cm height transferred to the greenhouse and grown in a mixture of commercial soil–sand–fertilizer mixture (Yrkes Plantjord, Kronmull, Weibulls Horto, Sweden) under 18-h photoperiod at 20/15°C (light/dark). In total, 25 trees per genotype were grown in the greenhouse. Trees were fertilized with about 150 ml of 1% Rika-S (N/P/K 7:1:5, Weibulls Horto) once a week. Trees were grown to about 0.8- to 1-m height with weekly rotation to minimize positional effects before 10 trees per genotype were inclined to an angle of 45° against a table and bound to that table approximately 10 cm below the tip of the stem for TW formation or kept upright for NW controls. After 22 days of inclination, stems were harvested between 10 and 50 cm above soil level; split into three parts for RNA extraction and transcriptomics, MFA measurements, and Fourier transform infrared (FT-IR) and Raman microspectroscopy; frozen in liquid nitrogen; and stored at −80°C until processing.

### Uplifting Experiments

After being outplanted into the greenhouse, wild-type and ETI trees were grown upright for 6 weeks. Then trees were either kept upright to continue forming NW, or the pots were horizontally inclined (90°) on the surface of a table to produce TW (see **Supplementary Video S1**). The uplifting response of the inclined trees was assayed over 4 weeks. All trees were grown in controlled greenhouse conditions with an 18/6-h photoperiod under light-emitting diode (LED lamps) (Fiona Lighting FL300 Sunlight), with an average day temperature ranging between 24°C and 25°C and average night temperature between 15°C and 17°C. Relative humidity ranged between 50% and 60%. Trees were fertilized once per week with Rika-S (Weibulls Horto, Hammenhög, Sweden), starting at the third week after planting and ending at the week before the experiments began in the case of the horizontally inclined trees, in order to reduce the additive effects on TW formation due to high nitrogen fertilization (Pitre et al., 2010). Measurements of height and diameter (twice, perpendicular at 10 cm above soil level) were taken weekly for upright grown trees, but only before inclination and after the end of the uplifting phase for the horizontally inclined trees. The stem spanning 5–12 cm above soil level was sampled in two parts. The lower half was frozen in liquid nitrogen and stored at −80°C, and the upper half was used directly for sectioning. Experiments were carried out on six biological replicates for each treatment and genotype. Time-lapse movies of the uplifting response were produced from photographs taken using a custom Raspberry Pi (Rpi) setup. Three cameras were connected to each Rpi, forming one unit. Units were clamped to the sides of opposing tables, and each camera was positioned to photograph two plants undergoing the gravibending response on the opposite tables. Using this setup, we captured 12 plants placed horizontally in each experiment. The Rpi was programmed to take photographs every 10 min the first day and thereafter once per hour for the remainder of the experiment. Quantification of stem lift and curvature was analyzed as described in Gerttula et al. (2015). In brief, we marked the point of primary bending with pink elastics (see in **Supplementary Video S1**). This point is defined as “the basal point of tropism of the primary, herbaceous portion of the stem” (Gerttula et al., 2015) and is clearly visible after a few hours of inclination. Thereafter, using the first picture taken each day (*n* = 28), we traced the stem from the base to the marked position (see in **Supplementary Video S1**). These stem traces were digitized in the form of *XY* coordinates in ImageJ (ImageJ, version 1.51j8 USA) and used to quantify the uplifting response. Normalized degree of lift was determined as the difference in height between the primary bending point and the base of the stem (in terms of their “*Y*” positions), divided by the respective length of the entire stem for each day [(*Y*_apex_ − *Y*_base_)/(Total Stem Length)]. We calculated the curvature (the summed differences in angle between subsequent pairs of *XY* coordinates or vectors), which indicates the deviation from a straight line in degrees. Scripts used in this experiment are available under https://github.com/UPSCb/UPSCb/tree/master/manuscripts/Seyfferth2019

### Histology, Microscopy, and Vessel Quantification

For both the upright and horizontally inclined trees, the stem at 10 cm above soil level was used to prepare fresh 70-µm-thick sections (vibrating blade microtome Leica VT1000S, Wetzlar, Germany) stained with Safranin : Alcian Blue (1:2) for 30 s, washed and mounted in 50% glycerol, and imaged with a Zeiss Axioplan2 microscope, AxioCam HRc camera, and AxioVision V 4.8.2 software (Carl Zeiss Light Microscopy, Göttingen, Germany). For TW : OW ratio determination, stereomicroscope images were obtained with a Canon PowerShot G7 digital camera. The distance from the center of the pith to the cambium of both the upper (TW) and lower (OW) sides was used to calculate the TW/OW ratio. Images for vessel quantification were taken with 10× magnification on a Zeiss Axioplan2 microscope. Images just inwards of the cambium, taken for NW, OW, and TW, were analyzed using ImageJ (version 1.51j8 USA).

### Vibrational Microspectroscopy and Data Analysis

FT-IR microspectroscopy with a single-element detector was performed using existing protocols (Gorzsás and Sundberg, 2014). Transverse sections with thickness of 20 µm were prepared from frozen stem samples using a Microm cryotome HM505E and dried in a desiccator between two polished rectangular BaF_2_ windows (38.5 mm × 19.5 mm × 4 mm, International Crystal Laboratories, Garfield, NJ, USA) for at least 48 h. One section from each of five biological replicates per genotype and treatment was analyzed. Single-element detector images were recorded over four positions, approximately 90° from one another around the entire cross section. A single spectrum was recorded from each position covering an area of approximately 430 × 430 µm, using a Bruker Equinox 55 spectrometer equipped with a Hyperion 3,000 microscopy accessory (Bruker Optics GmbH, Ettlingen, Germany). Spectra were recorded in transmission mode over the range of 700–3,800 cm^−1^, with a spectral resolution of 4 cm^−1^. Six hundred interferograms were co-added to improve signal-to-noise ratio, and a zero-filling factor of 2 was employed. Spectra were converted to data point tables using OPUS (v7.0.122, Bruker Optik GmbH, Ettlingen, Germany), baseline corrected (2-point linear baseline at 812 and 1,809 cm^−1^), and total sum (area-) normalized in the region of 812–1,809 cm^−1^, using in-house scripts (https://www.umu.se/en/research/infrastructure/visp/downloads/) written in Matlab (version 7.0, MathWorks, Natick, MA, USA, http://www.mathworks.com). Spectra were extracted, and the area measuring 816–1,803 cm^−1^ was used in subsequent multivariate analyses. Principal component analysis (PCA) and orthogonal projections to latent structures discriminant analysis (OPLS-DA) (Trygg and Wold, 2002) were performed using SIMCA-P+ (versions 12-14, Umetrics AB, Umeå, Sweden).

For Raman microspectroscopic analysis, 20-µm-thick transverse sections were prepared from frozen stem material (ca. 30 cm above soil level) using a Microm cryotome HM505E and kept between a standard glass microscope slide and cover slip in deuterated water (D_2_O). Spectral maps were recorded using a Renishaw inVia Raman spectrometer and microscope with a 100× oil immersion lens and a 514-nm Ar^+^ laser. Spectral maps comprising of 60–80 voxels with step sizes of 1 µm in the *X* and *Y* directions were recorded on two positions from each one to two cross sections of TW and NW, of each of three wild-type and three *pLMX5::etr1-1* trees that were grown upright or had been leaned at 45° and fixed in this position for 22 days. The maps were recorded in radial orientation over three fiber cells from lumen of cell 1 to lumen of cell 3. The following settings were used for spectral recording: static scans centered at 1,190 cm^−1^ (resulting in a spectral range of ca. 510 to 1,802 and ca. 1 cm^−1^ resolution with a 2,400 lines grating), standard confocality, laser power set at 100% in the software, and exposure time of 30 s per spectrum. Spectra were noise filtered and cleaned from cosmic rays using the chemometrics package of WiRE (version 3.4, Renishaw Plc, Wotton-under-Edge, UK). Data pre-processing was carried out in Matlab (version 2018a, MathWorks Inc., Natick, Massachusetts, USA), using an open source graphical user interface adapted from Felten et al. (2015) (https://www.umu.se/en/research/infrastructure/visp/downloads/) with the following parameters: spectra were cut to a range of 510 to 1,800 cm^−1^, asymmetric least squares baseline corrected (λ = 5,000, pVal = 0.001, Eilers, 2004), total area normalized, and smoothed with Savitzky–Golay filtering (first-order polynomial, with a frame size of 3). Multivariate curve resolution-alternating least squares (MCR-ALS) analysis was first conducted on TW samples. Based on singular value decomposition, the data were fitted using four components and only non-negativity constraints for both spectral and concentration directions. Two of the resolved components showed spectral features corresponding to bands previously assigned to cellulose and lignin. The third resolved spectral profile showed features characteristic of aromatic extractives, while the fourth had residual, unidentified contributions (**Supplementary Figure S1A**). These resolved spectral profiles were used as initial estimates in the MCR-ALS modeling of NW spectra (**Supplementary Figure S1B**). The resolved components were used to classify voxels into chemically distinct categories, using hard *k*-means clustering. Three clusters were used, corresponding to distinct zones: lumen (in all samples), G-layer and S-layer (in TW and NW samples, respectively), and S-layer plus middle lamella (in all samples) (**Figure 3**, **Supplementary Figure S2**). Spectral maps where no zones could be distinguished were removed from further analyses. Spectra originating from the distinct chemical zones of interest (cell wall layers) of the respective genotypes/conditions were used for PCA and, when appropriate, for OPLS-DA analysis (Trygg and Wold, 2002) in SIMCA-P+ (version 14.0, Umetrics AB, Umeå, Sweden) to reveal chemical changes in cell wall layers between the genotypes and conditions.

### Microfibril Angle Measurements

Radial-longitudinal stem sections of 100-µm thickness and 0.5- to 2-mm width were generated from six stems per genotype and condition (ca. 35 cm above soil level) using a Microm cryotome HM505E and dried between two microscopy glass slides. Xylem strips were transferred to metal holders for X-ray diffraction analysis. The X-ray diffraction experiments were carried out at the µ-spot beamline at the synchrotron facility BESSY II, Berlin, Germany. The radiation energy was set to 15 keV, corresponding to a wavelength of 0.8265 A. The (200)-Bragg peaks of cellulose, which were taken for orientation analysis, occurred at a scattering angle 2θ of 11.8. The samples were measured in ambient air, with the long axis of the fiber cells being perpendicular to the incident X-ray beam. The sample-detector distance was set to 245 mm, and the exposure time to 60 s. The beam diameter was set to 100 µm, so several measurements could be performed on one sample. Azimuthal intensity profiles (azimuthal angle ϕ vs. intensity) of the diffraction patterns were obtained by radially integrating the intensity of the (200)-peaks within 2θ ± 0.2° with an azimuthal step size of 1°. Microfibril orientation distributions and mean MFA were calculated by the simulation and fitting routine as described in detail previously (Rüggeberg et al., 2013).

### Transcriptomics and Data Analysis

**Figure 1:**
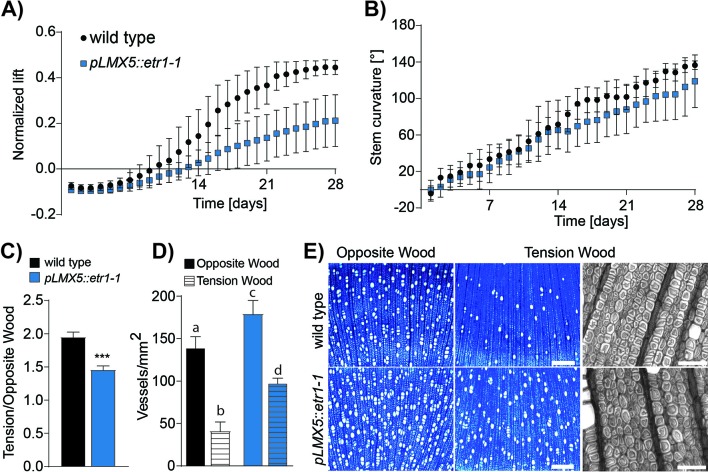
Quantification of the gravibending response in ETI hybrid aspen trees. **(A)** Normalized lift of six wild-type and six ETI trees (*pLMX5::etr1-1*). **(B)** Stem curvature of these trees. One time-lapse image per day was digitized to *XY* coordinates in ImageJ by tracing stems from the base to the point where primary bending occurs in the stem. Normalized degree of lift is the difference in height between the primary bending point and the base of the stem divided by the respective length of the entire stem for each day. Stem curvature is the summed differences in angle between each subsequent pair of *XY*. Error bars indicate ± SD. **(C)** TW : OW ratio of the ethylene-insensitive trees (*n* = 6). Asterisks indicate statistical significant differences from wild type according to Student *T*-test with Welch correction [pVal < 0.001 (***)]. **(D)** Vessel density in OW and TW of wild-type and ETI trees (*n* = 6). Error bars indicate ± SD. Letters indicate statistical significant differences between both genotypes and both tissues (OW/TW) calculated using a two-way analysis of variance (ANOVA) and a Tukey test (using a pVal cutoff = 0.05). **(E)** Representative 10× magnified images of OW and TW in inclined hybrid aspen wild-type or ETI stems. Insets show higher magnification of a TW area that visualizes the presence of G-layers in both genotypes. 10× magnified images were used for vessel density determination in **(C)**. Scale = 50 µm. ETI, ethylene insensitive; OW, opposite wood; TW, tension wood.

From greenhouse grown upright or inclined trees (22 days fixed at 45°) a 10- to 30-cm stem piece above soil level was harvested and frozen in liquid N2. We chose to analyze transcriptomes at a late stage of TW formation (22 days after inclination) since at this stage all processes that lead to uprighting are active, which is suggested by reduced performance in stem correction of ETI trees compared with WT (**Figure 1**, **Supplementary Video S1**). From trees forming TW, the bark was peeled off from the TW side of the stem, and developing wood and phloem/cambium (inner surface of the bark) were scraped with a scalpel into liquid nitrogen (Gray-Mitsumune et al., 2004). For NW, phloem/cambium and developing wood were scraped all around the stem after peeling off the bark. Scrapings from three trees per genotype and condition were pooled equally by weight and ground to a fine powder using a mortar and pestle in liquid nitrogen. One hundred milligram of powder was used to extract RNA using the cetyl trimethylammonium bromide (CTAB) method (Chang et al., 1993). DNA removal was carried out using DNA-free^™^ DNase (Ambion). After DNA removal, RNA was cleaned with the Qiagen MinElute kit. RNA concentration was measured with a Nanodrop ND-1000 (Nano-Drop Technologies, Delaware, USA), and its quality was assessed using an Agilent 2100 Bioanalyzer with Agilent RNA 6000 Nano Chips according to manufacturer’s instructions. Sequencing library generation and sequencing using Illumina HiSeq 2000 were carried out at SciLifeLab (Science for Life Laboratory, Stockholm, Sweden). Data sequences are available from the European Nucleotide Archive under the accession number PRJEB32252. Quality assessment and pre-processing of the obtained RNA-Seq reads were done following the guidelines described in Delhomme et al. (2014). Briefly, FastQC (v0.11.5; Andrews, 2010) was used to assess the quality of the raw data. Then, ribosomal RNAs and sequencing adapters were removed using SortMeRNA (Kopylova et al., 2012) and Trimmomatic (Bolger et al., 2014), respectively. Read quality was assessed using FastQC after both these steps and reads that passed the quality assessment were aligned to the *P. tremula* genome (v1; available at http://plantgenie.org/, Sundell et al., 2015). HTSeq was used for summarizing read counts (Anders et al., 2015). Read counts were normalized in R (v3.5.3, R Core Team, 2019) using DESeq2 (v1.30.0, Love et al., 2014) and used for PCA and differential gene expression analysis. Gene expression was normalized considering the effect from the genotype (wild type or *pLMX5::etr1-1*) and tissue (TW or NW) using the following model: gene counts ∼ genotype + tissue. Variance-stabilizing transformation was applied using the DESeq2 package. A PCA was conducted (using the plotPCA command in DESEq2) to show general differences between all analyzed samples. Differentially regulated genes (DRGs) were selected using an absolute log2 fold change (log2FC) larger or equal to 0.5 (pAdj < 0.05) as cutoff (suggested in Schurch et al., 2016). The script used for the transcriptome analysis is available under https://github.com/UPSCb/UPSCb/tree/master/manuscripts/Seyfferth2019. Gene ontology (GO) enrichment analysis was performed with the GO and PFAM enrichment tool at www.aspwood.org (Sundell et al., 2015). Visualization of comparative expression analyses was performed using the drawing tool available at http://bioinformatics.psb.ugent.be/webtools/Venn and gplots (v3.1.0). Promoter sequences of *Populus trichocarpa* of up to 2,000 bp in length were obtained and screened for the presence of the TEIL (tobaccoEIN3like) motif (AYGWAYCT; Kosugi and Ohashi, 2000), the GCC-box (AGCCGCC; Ohme-Takagi and Shinshi, 1995), and the ET-responsive element (ERE) (ATTTCAAA; Itzhaki et al., 1994). For precise promoter and cis-element prediction, we chose the *P. trichocarpa* genome (v3) as reference for this analysis, because it has a higher coverage in non-coding genome regions than does the available *P. tremula* genome sequence (v1). The “best hit” (*P. trichocarpa* sequence with the highest sequence similarity to *P. tremula* sequence) was defined as *P. trichocarpa* homolog, and the respective promoter sequences were obtained using the “Sequence Search” webtool from Popgenie (http://popgenie.org/sequence_search). Sequence match analyses are described in Felten et al. (2018).

### Quantitative Polymerase Chain Reaction (qPCR)

Total RNA extraction for qPCR analysis was carried out from 100-mg wood powder with Qiagen RNeasy Plant Mini kit according to the manufacturer’s instructions (using RLT buffer for lysis). Each of the three biological replicate samples per condition and genotype was a pool of xylem scrapings from three individual trees similar as described for material used for RNA-Seq experiments. An on-column DNA removal was carried out using Qiagen RNase-Free DNAse. Samples were subjected to a second DNA-removal step using the Invitrogen DNA-free kit according to the manufacturer’s manual. RNA quality was assessed by gel electrophoresis and quantitated by a Qubit 2.0 fluorometer using the Invitrogen Qubit RNA BR Assay. cDNA was synthesized using Biorad iScript from 300-ng RNA per sample according to the manual. qPCR was carried out on a Biorad CFX96 real-time PCR machine with a C1000 Touch Thermal Cycler in duplicate reactions measuring 15 µl consisting of 7.5 µl 2× Roche LightCycler 480 SYBR Green I Master solution, 0.3 µM of each primer, and 2 µl of cDNA. For evaluating primer efficiency, equal aliquots of every cDNA sample were taken, mixed, and diluted five times with a dilution factor of 5. For expression analysis, a 1/10 dilution of cDNA was used. The primer list is found in **Supplementary Table S8**. The qPCR program consisted of an initial denaturation step (95°C, 5 min); followed by 40 cycles of denaturation (95°C, 10 s), annealing (55–58°C, 10 s), and elongation (72°C, 20 s); and followed by a melting curve (65–95°C with a 0.5°C interval for fluorescence reading). Biorad CFX maestro software was used to determine the most stable reference genes among the five tested ones and yielded ideal values (*M* = 0.339 and stability Ln(1/Avg*M* = 1.082) for two genes, *UBC21* (*Potri.006G240900*) and *DAT* (*Potri.002G127700*), which were hence used for normalization of target gene expression data. Experimentally assessed primer efficiencies were taken into account for delta Cq calculations.

### Statistical Analysis

**Figure 2:**
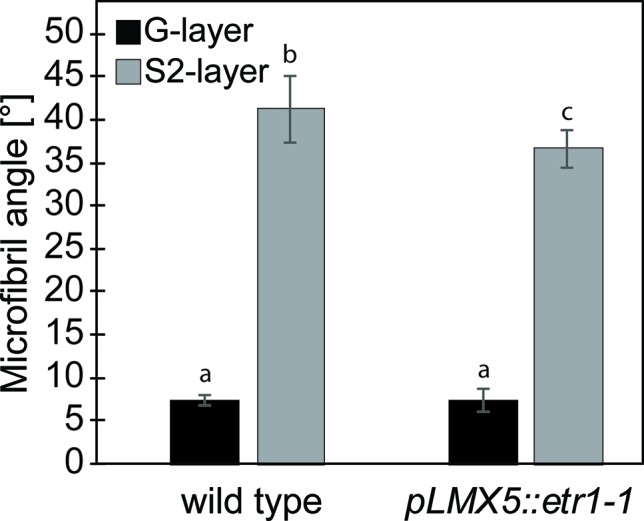
Cellulose microfibril angles in TW of hybrid aspen wild-type and ETI hybrid aspen trees (*pLMX5::etr1-1*). Bars represent average ± SD obtained from six individual trees per genotype. Letters indicate statistical significant differences between angles obtained in both genotypes and the G- or S2-layer calculated using a two-way analysis of variance (ANOVA) and a Tukey test (using a pVal cutoff = 0.05). ETI, ethylene insensitive; TW, tension wood.

**Figure 3:**
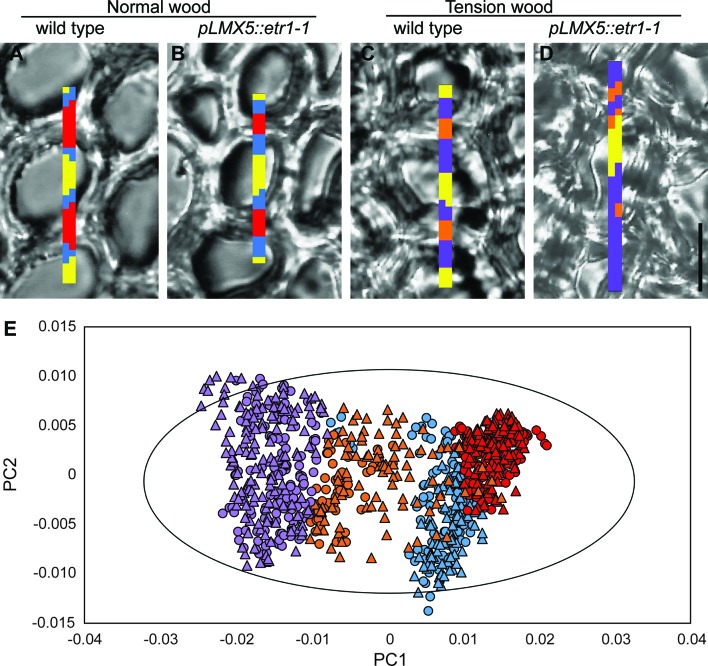
Identification and chemical comparison of cell wall layers in NW and TW of wild-type and ETI (*pLMX5::etr1-1*) hybrid aspen trees. **(A)** Overlay of white light images on one representative section out of six scanned sections per genotype and scanned maps after *k*-means clustering based on four MCR-ALS resolved components and three clusters. The sizes of the spectral maps are as follows: **(A)** 2 × 29 spectra, **(B)** 2 × 25 spectra, **(C)** 2 × 30 spectra, and **(D)** 2 × 34 spectra. Lateral step size is 1 µm^2^, and scale bar in **(D)** is 10 µm. Clusters in NW correspond to lumen (yellow), middle lamella + S-layer (red), and S-layer (blue); in TW, clusters correspond to lumen (yellow), middle lamella + S-layer (orange), and G-layer (violet). **(E)** Score plots (PC1 and PC2) of a PCA of spectra from all cluster maps shown in **Supplementary Figure S2**. Colors correspond to cell wall layers as described above. Triangles represent wild type, and circles *pLMX5::etr1-1.* Model details: autofitted (70 components, 893 observations), PC1 explained 79% of variation, PC2 explained 10% of variation. R2X(cum) = 0.996, Q2(cum) = 0.99. The ellipse in **(E)** indicates a 95% confidence interval (Hotelling’s *T* ^2^). ETI, ethylene insensitive; MCR-ALS, multivariate curve resolution-alternating least squares; NW, normal wood; PCA, principal component analysis; TW, tension wood.

**Figure 4:**
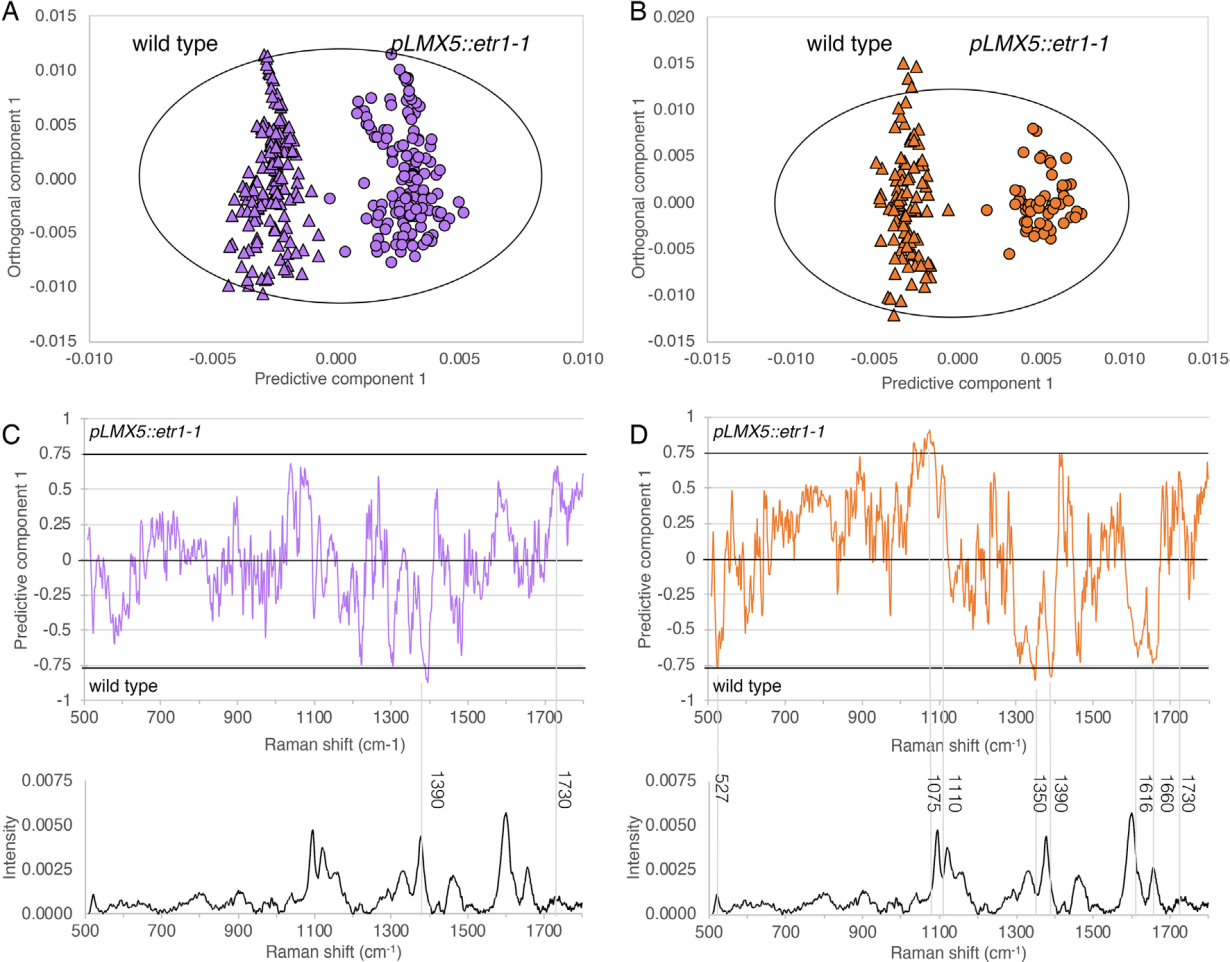
Chemical differences among G-layer **(A** and **C)** and S-layer **(B** and **D)** in TW of wild-type and ETI (*pLMX5::etr1-1*) hybrid aspen revealed by Raman microspectroscopy. **(A** and **B)** OPLS-DA score plots of 1 + 2 (predictive + orthogonal) component models based on the respective cluster extracted from all cluster maps shown in **Supplementary Figure S2**. Specification for the models: **(A)** 296 observations, R2X(cum) = 0.723, R2Y(cum) = 0.924, and Q2(cum) = 0.921; **(B)** 149 observations, R2X(cum) = 0.804, R2Y(cum) = 0.946, and Q2(cum) = 0.941. The ellipses in **(A)** and **(B)** correspond to 95% confidence intervals (Hotelling’s *T* ^2^). **(C)** and **(D)** The corresponding correlation-scaled loadings plots with a randomly selected spectrum underneath each representing the spectra used in the models. Wavenumbers with high correlation to separation are labeled. Noticeably, these do not always correspond to distinct peaks in **(D)** but can indicate shoulders (1,616 cm^−1^) or shifts towards different energy levels (lower, e.g., 1,075 cm^−1^; higher e.g., 1,390 cm^−1^), the latter being indicative of structural rather than proportional changes (qualitative rather than quantitative differences). ETI, ethylene insensitive; OPLS-DA, orthogonal projections to latent structures discriminant analysis; TW, tension wood.

**Figure 5:**
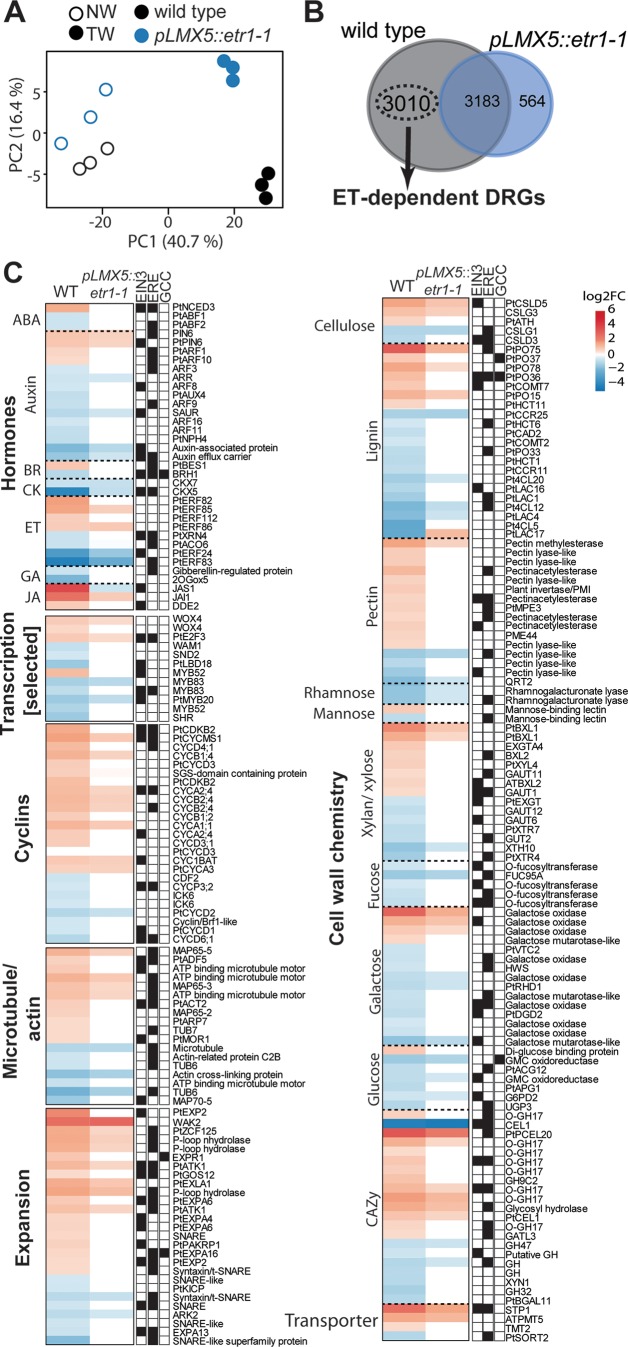
Transcriptome analysis between wild-type (WT) and ETI (*pLMX5::etr1-1*) hybrid aspen trees identifies ethylene-downstream genes with potential roles in TW formation. **(A)** PCA of transcriptome data. PC1 explains 41% of the variation and refers to the difference in NW and TW data. PC2 (16%) separates the TW transcriptomes of the WT and *pLMX5::etr1-1*. Each of the three replicate samples per genotype and condition originates from a pool of xylem/phloem scrapings from three trees. ERE, ethylene-responsive element; ETI, ethylene insensitive; NW, normal wood; PCA, principal component analysis; TW, tension wood. **(B)** Venn diagram highlighting the number of differentially regulated genes (DRGs; |log2FC| ≥ 0.5 and pAdj <0.05) in NW-to-TW comparisons. DRGs that only showed a significant change in expression in WT, but not in *pLMX5::etr1-1*, are further called “ET-dependent” (**Supplementary Tables S1–S3**). **(C)** Heat map showing expression changes (log2FC) of ET-dependent DRGs in TW compared with NW (**Supplementary Table S5**). Red indicates induction and blue suppression of gene expression in TW as compared with NW. White indicates absence of significant change in gene expression between TW and NW. Genes depicted in the heat map function in transcriptional regulation of cell division, xylem differentiation, and secondary cell wall biosynthesis; phytohormone signaling; microtubule and actin organization; and cell expansion and cell wall chemistry. Black boxes next to each gene indicate the presence of the ERE, TEIL-motif, and/or the GCC-box in its promoter region (**Supplementary Table S7**). ABA, abscisic acid; BR, brassinosteroid; CK, cytokinin; ET, ethylene; GA, gibberellins; JA, jasmonic acid.

Statistical analysis of OW/TW ratios (**Figure 1C**) between wild-type and ETI trees was calculated using a Student *T*-test with Welch correction. Differences were significant with pVal <0.05 (*), pVal < 0.01 (**), and pVal < 0.001 (***). Statistical differences between the number of vessels in TW and OW (**Figure 1D**) in wild-type and ETI trees were calculated by a two-way analysis of variance (ANOVA), and a post-ANOVA Tukey test was used for pairwise comparisons (pVal < 0.05). An ANOVA and a Tukey test were also used to assigned statistical differences in MFAs obtained in the G- or S2-layer and in gene expression of ET-dependent genes in wild-type and ETI trees (**Figure 2**). Details about PCA and OPLS-DA (Trygg and Wold, 2002) models generated in SIMCA-P+ (version 14.0, Umetrics AB, Umeå, Sweden) on spectroscopic data are given in the respective figure legends (**Figures 3** and **4**), and the choice of these methods is described in detail in Felten et al. (2018). Statistical analysis for differential gene expression analysis (**Figure 5**) was performed using pre-set statistical settings in the DESeq2 package (Love et al., 2014). Briefly, pValues were calculated using the Wald test, and multiple testing correction was performed using the Benjamini–Hochberg approach. Statistical tests for GO term enrichment were performed on the basis of Fisher’s exact test and included multiple testing (for detailed description, see Sjödin et al., 2009). Hypergeometric distribution was used to determine statistical significant enrichment of cis-elements (Felten et al., 2018; **Figure 5**).

## Results

### Ethylene Signaling Is Required for Fully Functional Tension Wood

We compared the uplifting response of wild-type and ETI hybrid aspen after horizontal inclination through time-lapse photography over a period of 28 days. The ETI trees overexpress the dominant negative *Atetr1-1* receptor under the control of the wood-specific *pLMX5* promoter. We have previously shown that transformation of hybrid aspen trees with the *pLMX5::etr1-1* construct conveys strong ET insensitivity to woody tissues (Love et al., 2009; Felten et al., 2018). Representative videos tracking the response of one out of the six tested wild-type trees and one out of the six ETI trees over the first 21 days of leaning were compiled using the same number of pictures per day and per genotype, enabling direct comparison (**Supplementary Video S1**). The videos illustrate qualitatively a delayed bending and reduced uplifting of the ETI trees as compared with the wild-type tree. To quantify this response, we selected the first time-lapse image of each day over the entire experimental period (*n* = 28) and calculated both the normalized lift and the curvature from stem traces captured in ImageJ (**Figures 1A, B**). While these two traits are interdependent, they also reveal slightly different aspects of the TW response since the same normalized lift may be supported by different curvatures. The curvature also gives an indication of the shape of the stem (e.g., 180° is a perfect half circle or “C” shape). The ETI trees showed a delayed bending response with a dramatic 52% reduction in lift relative to wild type on the final day of the experiment (**Figure 1A**). Stem curvature was also negatively affected in ETI trees, but to a lesser extent (**Figure 1B**). This illustrated that functional ethylene signaling is required in xylem tissues for the uplifting response.

### Asymmetric Growth and Vessel Reduction, but Not G-Fiber Formation, Require Functional Ethylene Signaling

To assess potential anatomical causes of the reduced uplifting response in ETI trees, we inspected the xylem anatomy in cross sections of leaned trees. The TW : OW ratio was significantly reduced in ETI trees (**Figure 1C**), confirming previous observations of reduced asymmetric growth in this genotype (Love et al., 2009). Horizontally inclined wild-type trees displayed a dramatic and expected decrease in vessel density on the TW side compared with the OW side after 4 weeks (**Figure 1E**). Compared with wild-type trees, the ETI trees had a higher vessel density in both TW (increase of 133%) and OW (increase of 30%) but not in NW (**Figures 1D, E**, **Supplementary Figure S3**). Strikingly, ETI trees presented G-fibers with no obvious anatomical difference compared with wild-type G-fibers, despite their reduced capacity to lift and the commonly held view of G-fiber involvement in generating the mechanical strain needed to uplift the stem (Mellerowicz and Gorshkova, 2012). Taken together, ETI trees showed decreased asymmetric growth, higher vessel density in both TW and OW, and a reduced uplifting response after inclination than did wild type, indicating that ET signaling is required for many characteristic features of the TW response, but not necessarily G-fiber formation.

### S-Layers in G-Fibers of Ethylene-Insensitive Trees Have an Altered Cellulose Microfibril Angle and Chemical Composition

With the aim to reveal potential causes of the attenuated uplifting response despite the presence of G-fibers in ETI TW, we inspected the cellulose MFA and chemical composition of the secondary cell wall more closely. When measuring MFA in TW of six wild-type and ETI trees that were each inclined to 45° and fixed in this position for 22 days, we observed in both genotypes the bimodal MFA distribution typical for TW and originating from small (0–5°) G-layer MFAs and larger (20–40°) S-layer MFAs (Müller et al., 2006; Goswami et al., 2008; Rüggeberg et al., 2013). This was expected as both genotypes formed S2- and G-layers (**Figure 1E**). There was no significant difference in average G-layer MFAs between the genotypes (**Figure 2**). However, the average S2-layer MFAs in ETI trees were significantly lower than those in wild-type trees (36.7 ± 2.2° for ETI trees and 41.2 ± 4° for wild-type trees).

We furthermore assessed whether G-fiber-rich TW areas were chemically altered, in cross sections of ETI and wild-type trees using FT-IR microspectroscopy with a single-element detector. PCA performed on these spectra did not show clear grouping (**Supplementary Figure S4**), indicating that the overall chemical composition of the sampled areas is not altered. As unaltered cells/cell wall layers can mask subtle changes confined to small zones/specific layers, we used Raman microspectroscopy with high lateral resolution to specifically focus on the chemical information from G- and S-layers in wild-type and ETI trees. While analyzing data from spectral maps scanned over three adjacent fiber cells with 1-µm step size in TW and NW of both genotypes, four chemically different zones were assigned based on spectral information that overlapped with cell layers visible in the sections: (1) cellulose rich G-layer, (2) cellulose- and lignin-containing S-layer, (3) lignin-rich zone including both the S-layer and the middle lamella (hereafter called S + ML), and (4) lumen (**Figures 3A–D**, **Supplementary Figures S1, S2**). Comparison of NW S-layer spectra between wild-type and ETI trees or NW S + ML layer spectra between wild-type and ETI trees by PCA did not reveal any clear grouping and thereby ruled out any genotype-related chemical differences at the start of the leaning experiment (**Supplementary Figure S5**). PCA of non-lumen spectra from TW and NW from both genotypes revealed clear groupings of TW and NW samples (**Figure 3E**) as well as between the respective cell wall layers. This suggests that TW is chemically different from NW not only by the addition of the G-layer to fibers but also by a chemical alteration of the S-layer itself. The respective OPLS-DA models for TW G-layer and TW S-layer spectra of both genotypes indicated a chemical difference in both layers between wild-type and ETI trees (**Figures 4A, B**). The corresponding correlation-scaled loadings revealed a limited number of distinct bands that contribute to the difference in the G-layer (**Figure 4C**), a higher proportion of –C═O double-bond vibrations in the ETI G-layer (1,730 cm^−1^, not related to cellulose), together with a lower proportion of –C–H vibrations (1,376 cm^−1^, typically assigned to cellulose based on Gierlinger and Schwanninger, 2006). The correlation-scaled loadings for S-layers in G-fibers revealed more variables contributing to the differences between wild-type and ETI trees (**Figure 4D**). The S-layer spectra in ETI G-fibers had, in addition to bands already marked in the G-layer loadings in **Figure 4C**, a lower proportion of lignin-associated vibrations (1,616 and 1,660 cm^−1^) and a higher contribution of bands associated with polysaccharidic compounds (carbohydrate ring-associated vibrations around 1,100 cm^−1^). Taken together, Raman microspectroscopy was able to identify chemical differences among S- and G-layers of wild-type compared with ETI TW, with a clearer difference in the S-layers of G-fibers, as compared with their G-layers. This is in line with results from X-ray diffraction that also revealed a stronger effect on the S-layer ultrastructure of the G-fibers as compared with the G-layers themselves in ETI trees.

### Ethylene Signaling Suppresses Lignin Biosynthesis Genes and Induces Expression of Microtubule and Actin-Related Genes in Tension Wood

To understand the impact of ET signaling on transcriptional reprogramming during TW formation, we compared transcriptomes from TW (trees inclined to and fixed at 45° for 22 days) and NW (upright trees) forming tissues in wild-type and ETI trees (**Figure 5**). PCA suggested that the predominant difference between the obtained transcriptomes (describing 41% of variation in the data set) is explained by the tissue type (NW or TW) (**Figure 5A**). The second component (describing approximately 16% of the overall variation in the data set) separated transcriptomes based on the genotypic background. The separation between wild-type and ETI trees was seen for both TW and NW transcriptomes. We next selected genes with significantly changed expression (DRGs, |log2FC| ≥ 0.5 and pAdj < 0.05) in TW compared with NW in both the wild-type and ETI trees (**Supplementary Tables S1, S2**). We detected 6,193 DRGs in wild type, while only 3,747 DRGs were identified in ETI trees (**Figure 5B**). We observed an almost equal distribution of induced and repressed genes in both genotypes (**Supplementary Figure S6A**). Expression of 3,010 DRGs, with close to equal numbers of induced and repressed ones, was significantly changed only in TW of wild-type trees (**Figure 5B**, **Supplementary Table S3**). Loss of transcriptional regulation of these genes in the ETI trees implicates that their expression requires functional ethylene signaling. We therefore defined these genes as “ET-dependent genes.” GO term analysis of the ET-dependent genes revealed an enrichment of genes related to ribosomes and translation (**Supplementary Table S4**). Functional characterization of the ET-dependent genes identified several TFs with known functions in xylem differentiation and gene networks related to hormone pathways, cell expansion, cytoskeleton organization, and cell wall chemistry (**Figure 5C**, **Supplementary Table S5**). To investigate a potential direct transcriptional regulation of these ET-dependent genes by TF families that function downstream of the ET receptors, we screened promoter regions of ET-dependent genes (2,000-bp upstream of the start codon) for the presence of the ERE, EIN3 (TEIL)- and ERF (GCC-box)-binding motifs (**Figure 5C**, **Supplementary Table S6**). The vast majority of the selected ET-dependent genes with a function in wood development, anatomy, or chemistry contained the ERE motif (90 genes), the TEIL motif (66 genes), and/or a GCC-box (six genes) in their promoter region. Approximately 37% (101 genes) of the 234 ET-dependent genes that were selected based on their association with hormone pathways, cell expansion, cytoskeleton organization, cell wall chemistry, or transcriptional regulation during wood formation did not contain any motif.

We detected 35 hormone-related genes among the ET-dependent DRGs, with the largest group of them (15 genes) being related to auxin transport and signaling (**Figure 5C**). The ET-dependent genes suggested that ET signaling stimulates induction of abscisic acid (e.g., *9-CIS-EPOXYCAROTENOID DIOXYGENASE* (*PtNCED*)) and jasmonic acid (e.g., *DELAYED DEHISCENCE 2* (*DDE2*) biosynthesizes genes and suppresses genes involved in ET biosynthesis (e.g., *PtACO6*) and degradation of cytokinins (e.g., *CYTOKININ OXIDASE*s (*CKX*s)). We also identified genes involved in signaling of auxin (e.g., *AUXIN RESPONSE FACTOR*s (*ARF*s)), brassinosteroids (e.g., *BRI1-EMS-SUPPRESSOR 1* (*BES1*)), and gibberellins (GAs) (e.g., *A-STIMULATED IN ARABIDOPSIS 14* (*GASA14*)). We evaluated the effect of overexpression of *Atetr1-1* on the expression of genes related to the ET pathway (**Supplementary Figure S6B**). Whereas TW formation resulted in differential expression of ET biosynthesis genes (*PtSAM*s, *PtACO*s, and *PtACS*) regardless of ET insensitivity, differential expression of many ET downstream genes (mainly TFs like *PtEIN3* and various *PtERF*s) was absent or attenuated in ETI trees during TW formation. We further found that wild type, but not ETI trees, showed induced expression of genes involved in cell division (*WOX4* (*WUSCHEL RELATED HOMEOBOX 4*), *E2F3* (*E2F TRANSCRIPTION FACTOR 3*), and *CYCLIN*s), actin and microtubule organization (e.g., *MICROTUBULE-ASSOCIATED PROTEINS* (*MAP*s)), and cell expansion (e.g., *EXPANSIN*s) in TW (**Figure 5C**; **Supplementary Figure S6D**). The ET-dependent genes also included two key TFs for xylem fiber differentiation and vascular patterning, *SND2* (*SECONDARY WALL-ASSOCIATED NAC DOMAIN PROTEIN 2*) and *SHR* (*SHORT ROOT*). These TFs were suppressed in TW of wild-type trees, while no change in expression was observed in ETI trees. In line with the observed lower amount of vessels in TW of wild-type trees (**Figure 1D**), we observed during TW formation in wild-type trees a slight repression of two *Populus* homologs (*PtVND7.1* and *PtVND7.2*) of the master regulator of vessel formation *VASCULAR-RELATED NAC-domain 7* (*VND7*) (although statistically not significant, **Supplementary Figure S6C**). During TW formation in ETI trees, no transcript change was observed for *PtVND7.2* but a slight, but not statistically significant, induction of *PtVND7.1*. Statistically significant ET-mediated suppression of genes involved also genes in the lignin pathway (e.g., *MY52*, *MYB83*, *CAFFEATE O-METHYLTRANSFERASE2* (*PtCOMT2*), and *CINNAMYL ALCOHOL DEHYDROGENASE* (*PtCAD2*)) (**Figure 5C**), whereas no phenylalanine ammonia lyase (PAL)-encoding genes that act upstream during lignin biosynthesis were among our target genes. qPCR results validated that ETI trees lost regulation of genes involved in lignin biosynthesis such as *PtCOMT2* during TW formation (**Supplementary Figure S6D**). The ET-dependent genes also comprised genes encoding cellulose synthases (e.g., *PtCSLD5*), pectin lyases/acetyltransferase/methylesterases (e.g., *PME44*), CAZymes (e.g., *CELLULASE1*), and sugar transporters (e.g., *SUGAR TRANSPORTER1* (*STP1*)). Despite the large impact of ET on gene regulation in TW, only two G-layer-associated fasciclin-like arabinogalactan (*FLA*) genes (Lafarguette et al., 2004; Kaku et al., 2009) were found among the ET-dependent genes (*FLA1* and *AGP14*, **Supplementary Table S3**), which is in line with the presence of a G-layer even in ETI trees (**Figure 1E**).

This analysis of ET-dependent genes suggests that ET signaling triggers expression of genes underlying cell division and expansion and suppresses genes involved in vessel differentiation and biosynthesis and polymerization of lignin. A part of these genes may be directly regulated by TFs of the ET signaling cascade as indicated by the presence of GCC-box, TEIL, or ERE motifs in the promoters of these genes.

## Discussion

**Figure 6:**
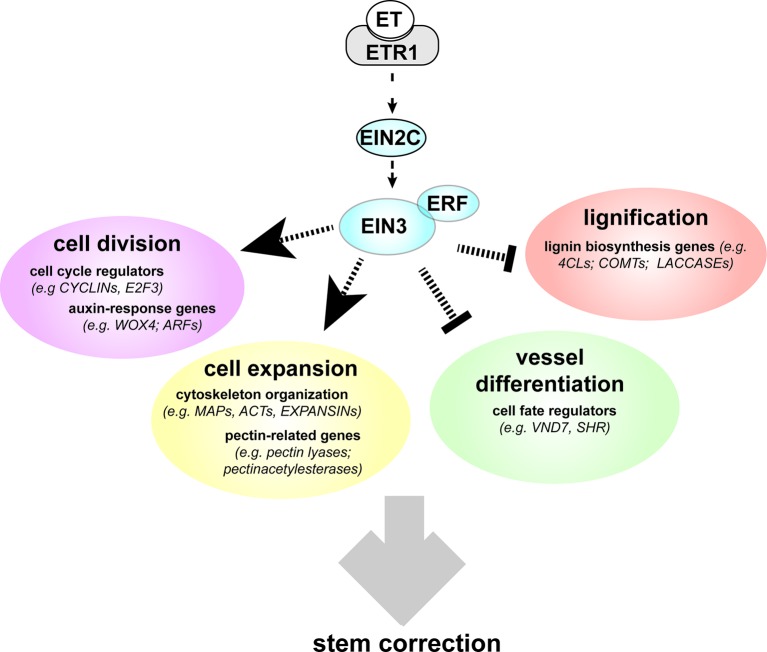
Model showing ethylene-regulated pathways that potentially induce anatomical, ultrastructural, and chemical modifications of xylem cells during TW formation. Ethylene stimulates cell division during TW formation possibly by EIN3 binding to the promoter of *PtE2F3*, by induction of *PtWOX4* through ethylene–auxin interaction and by direct or indirect induction of *PtCYCLIN*s. Ethylene suppresses vessel formation and lignification (likely because of the decreased amounts of highly lignified vessels) during TW formation. Vessel suppression might be mediated by *PtVND7* repression, through PtE2F3, and repression of *SHR*, potentially through ethylene–auxin interaction. Suppression of lignin biosynthesis is likely a result of a combination of a direct gene regulation of lignin genes with ERE, TEIL, and GCC-boxes in their promoter, as well as a secondary effect resulting from suppression of vessel differentiation. During TW formation, ethylene alters gene expression related to cell wall expansion, such as expansins *PtEXP*s, *PtMAP*s, and pectin-related genes. Expansin induction goes in line with enhanced primary wall formation due to enhanced cambial activity and cell proliferation during TW formation. *PtMAP*s could influence the cytoskeleton and subsequently cellulose microfibril angles. Direct regulation through EIN3 and ERFs can be assumed for *EXPANSIN*s harboring TEIL and GCC-boxes in their promoter as well as indirect effects through ethylene–auxin interaction. TW, tension wood.

Understanding the mechanisms by which trees sense displacement and react to correct their stem position through TW formation is a long-standing question. A role for phytohormones, such as GAs, auxin, and ET, has been discussed in this context (Andersson-Gunnerås et al., 2003; Love et al., 2009; Nugroho et al., 2012; Gerttula et al., 2015), but the significance of ET signaling remains unclear. In this study, we report on the importance of signaling by the phytohormone ET for both TW formation and functionality. ET signaling is often induced in response to environmental stimuli such as abiotic and biotic stresses (Dubois et al., 2018). In the absence of any stress signals, such as during upright growth in greenhouse conditions, ETI trees are similar to wild-type trees and do not show any obvious growth defects (Love et al., 2009). Therefore, these trees are ideally suited to study the role of ET signaling during an induced stress like displacement. Our data indicate that loss of ET signaling in ETI trees restricts the lifting response of inclined stems (**Figure 1**) and interferes with most developmental responses that are characteristic for TW, but not the formation of G-layers. Stem eccentricity observed in TW is the result of enhanced cambial growth at the upper side of the leaning stem (Ruelle et al., 2006; Ruelle, 2014). Stimulation of xylem growth on the TW side was attenuated in ETI trees during tilting resulting in a lower TW : OW ratio than in wild-type trees. The concept that the reduced TW : OW ratio results from reduced cambial cell division in ETI TW (**Figure 1C**) is supported on a molecular level by the attenuated reprogramming of cell cycle regulators, such as *CYCD3* and *E2F3* in ETI trees during TW formation (Kosugi and Ohashi, 2002), as well as regulators of cambial cell division activity like *WOX4* (Hirakawa et al., 2010; Ji et al., 2010; Suer et al., 2011; Kucukoglu et al., 2017; Shi et al., 2019; **Figure 5C**). *WOX4* expression is controlled by auxin signaling. Auxin forms a gradient over the cambium in the stem and is known to function as a positional signal and to positively influence cambial growth (Tuominen et al., 1997; Uggla et al., 1998; Smetana et al., 2019). Conversely, inhibiting auxin signaling in stems of transgenic trees negatively impacts (in addition to primary also) secondary growth (Nilsson et al., 1992). We identified several genes related to auxin transport and signaling (e.g., *PIN6* and *ARF*s) among the ET-dependent DRGs in our study (**Figure 5C**), which is in line with previous reports that showed that ET influences both auxin biosynthesis and transport (Swarup et al., 2007; Muday et al., 2012; Vaseva et al., 2018). Based on these results, it is possible that the reduced stimulation of cambial growth observed in ETI TW is explained through ET–auxin crosstalk. ETI TW showed a reduced amount of G-fibers than did wild-type TW, due to the mentioned reduced cambial stimulation and as a result of lower fiber-to-vessel ratio because of reduced suppression of vessel formation (**Figure 1C**; Love et al., 2009). Reduced vessel frequency is observed in many other species during TW formation (Yoshizawa et al., 2000; Jourez et al., 2001; Ruelle et al., 2006; Sultana et al., 2010). Results from yeast one-hybrid assays identified the cell-cycle regulator E2Fc as a regulator of transcriptional gene networks for secondary cell wall formation in *Arabidopsis*. E2Fc can directly bind to promoter regions of NAC TFs involved in vessel differentiation, namely, *VND6* and *VND7* (Taylor-Teeples et al., 2015). Only TW of wild-type trees showed induction of a *Populus E2F* homolog (*E2F3*) and a slight, yet not statistically significant, suppression of *PtVND7.1* and *PtVND7.2*, while such expression changes were not observed in ETI trees (**Figure 5C**, **Supplementary Figure S6C**, **Supplementary Tables S3, S7**). Furthermore, *PtLBD18* (*LATERAL ORGAN BOUNDARIES DOMAIN 18*), a homolog of *AtLBD18*, which has been shown to regulate expression of *AtVND7* (Soyano et al., 2008), was among the ET-dependent genes. The EIN3-binding motif is present in promoters of *Populus E2F3*, *LBD18*, and *VND7*, suggesting that expression of these genes might be under the direct control of EIN3 during TW formation (**Figure 6**). Furthermore, ET–auxin interaction might also contribute to the changes in vessel formation. Auxin, when exogenously applied, suppresses expression of *NAC*s involved in fiber differentiation and stimulates expression of *NAC*s involved in vessel formation in hybrid aspen stems (Johnsson et al., 2018). Expression of several genes involved in auxin signaling was regulated by ET in TW (**Figure 5C**), some of them harboring TEIL or ERE motifs in their promoters (e.g., *PtPIN6*, *PtARF1*, and *PtARF10*). Therefore, in addition to a direct effect of ET on *NAC* expression and vessel density, a potential regulation of vessel differentiation through *NAC* regulation *via* ET–auxin crosstalk is also plausible. The absence of repression of *E2F3* and *VND7* in ETI TW could also contribute to the reduced suppression of vessel development during TW formation in ETI trees. The reduction of vessel elements in TW of wild-type trees as compared with NW likely causes an absolute decrease of lignin levels on tissue level, because lignin content in vessel cell walls is generally higher as compared with the one in fiber S2 walls (Saka and Goring, 2009; Xu et al., 2005). As less of the highly lignified vessels are formed in wild-type TW compared with NW, the lignin biosynthesis machinery is expected to be downregulated. During formation of TW in ETI trees, the absence of vessel suppression goes in line with an absence of downregulation of genes involved in lignin biosynthesis as observed in our data (**Figure 5C**). An effect of ET signaling and regulation of lignification has previously been demonstrated in experiments where lignification of tracheary elements (TEs) in cell culture was blocked by inhibiting ET signaling (Pesquet et al., 2013). For a few of the ET-dependent lignin biosynthesis genes from our data set, we identified the EIN3, GCC-box, or ERE-motif in their promoters, suggesting a direct regulation of their expression by TFs in the ET pathway (**Figures 5C** and **6**).

We previously reported that exogenously enhancing ET signaling was sufficient to induce the G-layer formation in hybrid aspen in an ET signaling-dependent manner (Felten et al., 2018). Unexpectedly, in the present study, we observed that G-fibers with G-layers could form during TW formation in ETI trees (**Figure 1E**), despite inhibited ET signaling, with no altered ultrastructure in the G-layer (**Figure 2**) and no dramatic changes in gene expression typically associated with G-layer presence, such as induction of *FLA*s (**Supplementary Table S2**). This is, to a large extent, similar to what has been found with GAs. Although it remains to be shown that GA biosynthesis is altered during tilting, exogenously applied GAs can induce G-fiber formation and xylem growth (Jiang et al., 1998; Jiang et al., 2008; Nugroho et al., 2012). Conversely, inhibition of GA signaling counteracts stem bending and lifting and lowers eccentric growth in displaced stems, despite G-fibers being formed (Jiang et al., 1998; Jiang et al., 2008; Nugroho et al., 2012). This suggests that both GA and ET signaling can induce G-fiber formation but that inhibiting one of these pathways does not abolish G-fiber formation. We found very few GA-associated genes among the ET-dependent DRGs, suggesting that GA signaling is not impacted in ETI trees. It will be interesting to investigate whether a combination of both ET and GA signaling inhibition can fully inhibit the G-layer formation during stem tilting and whether these phytohormone pathways can compensate for each other’s absence. The present study also confirms what was observed with exogenous ACC/ET application experiments (Felten et al., 2018), that the effect of ET signaling on vessel and the G-layer formation is uncoupled and must therefore occur as independent routes downstream of ET signaling.

Even though G-fiber formation as such was not inhibited during TW formation in ETI trees, we observed ultrastructural and chemical alteration of G-fiber S-layers in ETI TW (**Figures 2**–**4**). The significantly lower MFA of the S2 layer in ETI TW suggests a role of ET in modification of secondary cell wall MFA in TW. G-layers had, however, no alternated MFA in ETI TW. Using Raman microspectroscopy, we observed chemical alterations in both the G- and S-layers of G-fibers as a result of ET insensitivity (**Figure 4**). Our spectroscopic data suggest differences both in the relative proportions of major cell wall polymers and in the structure of the polymers such as changes in cellulose crystallinity or a difference in cross-linking of polymers. It is possible that the chemical differences identified originate either from the layers itself or from a shift in clustering related to the composition of layer boundaries (e.g., the boundary of S- and G-layers). Gradients across the G-layer in both the polymer composition and the cellulose ultrastructure have been observed (Prodhan et al., 1995; Joseleau et al., 2004; Gierlinger and Schwanninger, 2006; Arend, 2008; Sandquist et al., 2010; Ruelle, 2014). At the S-G-layer interface, an accumulation of xyloglucan and rhamnogalacturonan has been detected (Sandquist et al., 2010) and linked to the mechanisms required for stress generation in G-fibers (Nishikubo et al., 2007; Mellerowicz and Gorshkova, 2012). As such boundaries and gradients are narrower than what can be resolved in a separate class by the Raman microspectroscopy, their chemical composition will influence the cluster that they are included in by *k*-means clustering. If the composition of this boundary was altered in ETI TW, the pixels comprising them may chemically become more similar to another neighboring layer (S instead of G) and therefore be included in that latter cluster. In conclusion, the chemical differences detected in the ETI G-fiber S-layer (lower lignin and higher polysaccharide contribution) could result either from a chemical alteration within the layer or from a higher proportion of S–G-boundary pixels, low in lignin and high in hemicellulose, included in the cluster of this layer. This is, to our knowledge, the first report that shows that ET can interfere with both the cellulose ultrastructure and the chemical composition of the secondary cell wall.

In summary, we have detected both ET-dependent quantitative (G-fiber number) and qualitative (G-fiber properties) aspects that could contribute to the observed attenuated uplifting in ETI plants. The G-layer itself does not seem to be a prerequisite for high tensile stress generation in TW, since other cell wall layer arrangements leading to a low cellulose MFA in the main cell wall layer can also induce the tensile stress required for stem lifting (Okuyama et al., 1994; Yoshizawa et al., 2000; Ruelle et al., 2007a; Ruelle et al., 2007b). However, in species containing a G-layer, there is a relationship between its thickness, its cellulose MFA, and the potential growth strain exerted (Fang et al., 2008; Goswami et al., 2008). Typically, in TW of *Populus*, the MFA in the S-layer is much larger than in NW, while the G-layer inherits an MFA close to 0° (Timell, 1986; Lautner et al., 2012). It has been hypothesized that the interplay between the G-layer and the S2-layer with its high MFA facilitates generation of the tensile stress upon swelling, required to correct the stem position through uplifting (Goswami et al., 2008). G-layer swelling leads to a pressure on the surrounding cell walls that is translated into axial stress. According to a model by Goswami et al. (2008), this stress enhancement factor depends on the orientation of the MFAs in the S2 layer. The MFA alteration observed in our study for the S2-layer of ETI TW is within the range of stress enhancement factors leading to a sufficient axial stress for uplifting and lies in a range of MFAs also observed in TW of other wild-type trees with G-fibers. Taken together, as the G-layer MFA is not altered in ETI TW and the S-layer MFA is not altered to an extent that would limit the uplifting response according to the model proposed by Goswami et al. (2008), the origin of inhibited lifting observed in ETI trees (**Figure 1**) can not only be attributed to altered MFA angles. Other authors have observed a correlation between the stem bending response and the amount of G-fibers formed (Nugroho et al., 2012). This indicates that there is both qualitative and quantitative effects of G-layers/G-fibers on the potential of stem reorientation. Given the rather low changes in qualitative G-fiber properties in ETI TW in comparison with the large effect on G-fiber quantity, we propose that it is rather the latter that is decisive for the reduced uplifting response, even though we cannot exclude that G-fiber amounts and S-layer chemistry and ultrastructure in ETI G-fibers have additive effects.

Taken together, the results presented here demonstrate the importance of ET signaling to define TW characteristics and to correct stem position after tilting. We also revealed key genes that potentially can coordinate these responses downstream of ET signaling during TW formation (**Figure 6**).

## Data Availability

The raw sequencing data for this study can be found in the European Nucleotide Archive (ENA) under accession PRJEB32252.

## Author Contributions

CS performed transcriptomic analysis. BW performed and analyzed uplifting experiments. AG performed FT-IR and Raman microspectroscopic measurements and data analysis. JL supplied biological material for FT-IR measurements. MR carried out cellulose MFA measurements. ND gave support for transcriptomics analysis. TV and BW performed vessel density quantification. KA developed software for data analysis of uplifting experiments. BS and JF conceived the study. JF, BS, and HT supervised the experiments. JF generated biological material for the experiments. CS and JF wrote the manuscript with contribution of all authors.

## Funding

The experiments as well as BW, JWL, and JF were funded by the Swedish Research Council Formas (grant nos. 213-2011-1148 and 239-2011-1915) and the Swedish Foundation for Strategic Research (grant no. RBP14-0011). CS was funded by The Kempe Foundation (SMK-1649 and SMK-1533) and the Lily och Sven Lawskis fund. Experimentation was financially supported also by the Swedish Governmental Agency for Innovation Systems Vinnova (grant no. 2016-00504) and the Knut and Alice Wallenberg Foundations (grant no. 2016-0341).

## Conflict of Interest Statement

Björn Sundberg is employed by Stora Enso AB, Sweden. The remaining authors declare that the research was conducted in the absence of any commercial or financial relationships that could be construed as a potential conflict of interest.

## Supplementary Material

Supplementary Video S1 Time-lapse video of the uplifting response of one representative pLMX5::etr1-1 and one representative wild type hybrid aspen tree imaged over the first 21 days of the experiment.
