# Supplementary material for: Ethylene Signaling Is Required for Fully Functional Tension Wood in Hybrid Aspen
Source: Front Plant Sci. 2019 Sep 26;10:1101. doi: 10.3389/fpls.2019.01101 (PMC6775489; doi:10.3389/fpls.2019.01101)
Supplement: Supplementary file 2 [file DataSheet_1.pdf]

Supplementary figures

Ethylene signaling is required for fully functional tension wood in hybrid aspen

Carolin Seyfferth , Bernard A. Wessels, András Gorzsás , Jonathan W. Love, Markus Rüggeberg , Nicolas Delhomme, Thomas Vain, Kamil Antos , Hannele Tuominen, Björn Sundberg, Judith Felten

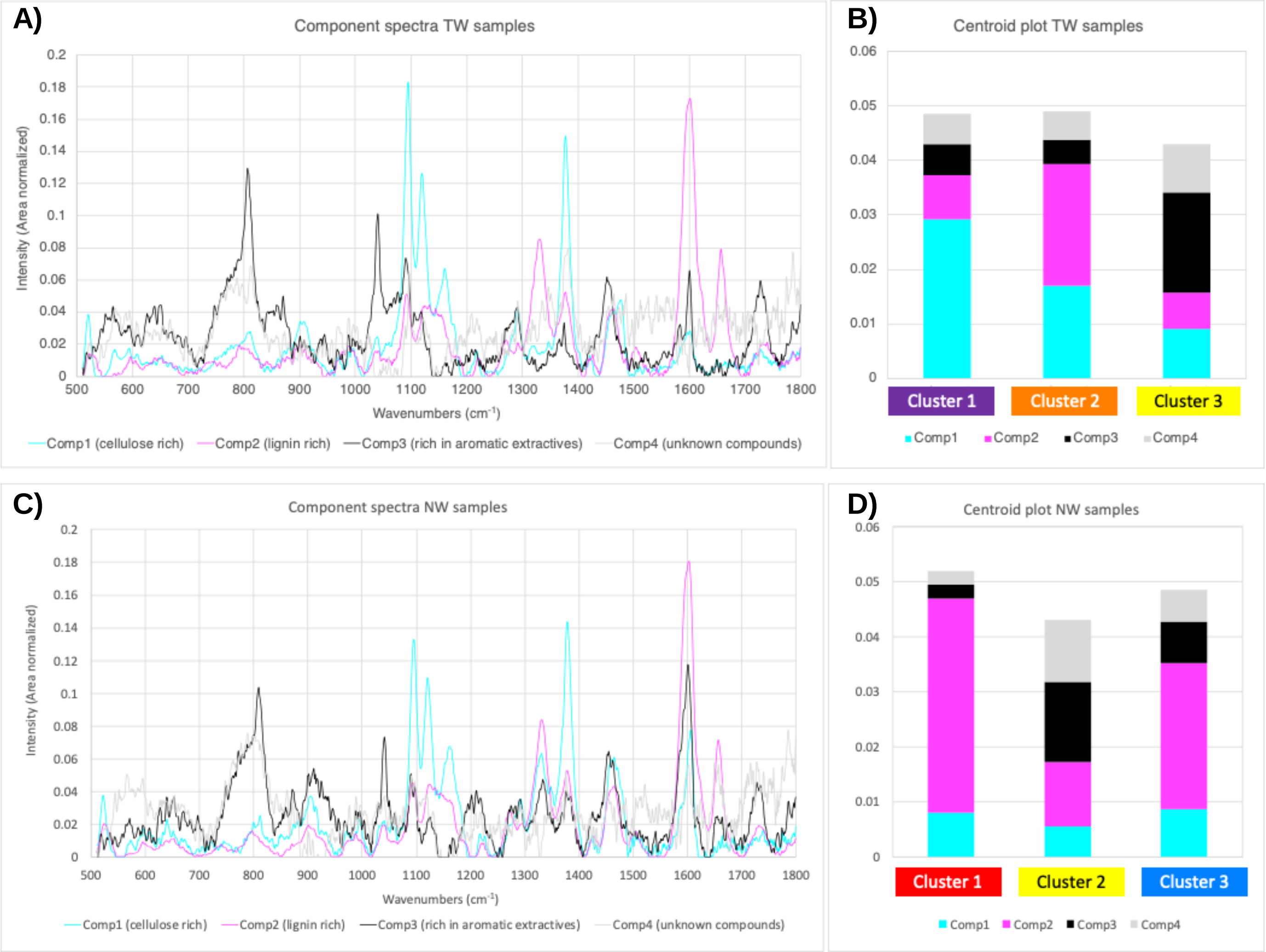

Supplementary Figure S1: Component maps resolved from TW samples (A) and NW samples (C) using MCR-ALS as well as centroid plots for TW samples (B) and NW samples (D) following k-means clustering. Four components were resolved in TW and used as initial estimates to resolve the corresponding four components in NW. Component 1 displays bands characteristic for polysaccharides, and in particular cellulose (around 1100-1165 cm-1, 1376 cm-1), Component 2 is dominated by bands characteristic for lignin (1600 cm-1, 1660 cm-1), Component 3 contains bands resembling those of aromatic extractives (810 cm-1, 1030 cm-1, 1600 cm-1), whereas diagnostic bands in Component 4 could not be identified. Comparing Component 1 in NW and TW reveals different bands ratios, indicating lower crystallinity of cellulose in NW as compared to TW. Centroid plots indicate the contribution of the respective components to the clusters identified in the Raman maps (Figures 3A-D and Supplemental Figure S2). Cluster 1 in TW has a high contribution from Component 1 (the polysaccharide/cellulose rich component) and represents the G-layer; Cluster 2 in TW has significant contributions form both Components 1 and 2 (polysaccharides/cellulose and lignin) and indicating spectra originating from S-layer/middle lamella; Cluster 3 represents the lumen (with the highest contribution of Components 3 and 4, i.e. extractives and noise). In NW, Cluster 1 is dominated by contributions from the lignin rich Component 2 with some contribution from the polysaccharide/cellulose rich Component 2, corresponding to the lignin-rich middle lamella with potential contribution from neighbouring layers; Cluster 2 contains higher contributions from noise and extractives (Components 4 and 3, respectively), thus describing the cell lumen; Cluster 3 contains lower contributions from the lignin-rich Component 1 as compared to Cluster 1, but otherwise similar to Cluster 1, therefore referring to S-layer, as validated by its distribution / location within the images. Colours on the bars represent the Component colours used in A and C, while the colours on the cluster names are the ones used in the cluster maps shown in Supplementary Figure 2.

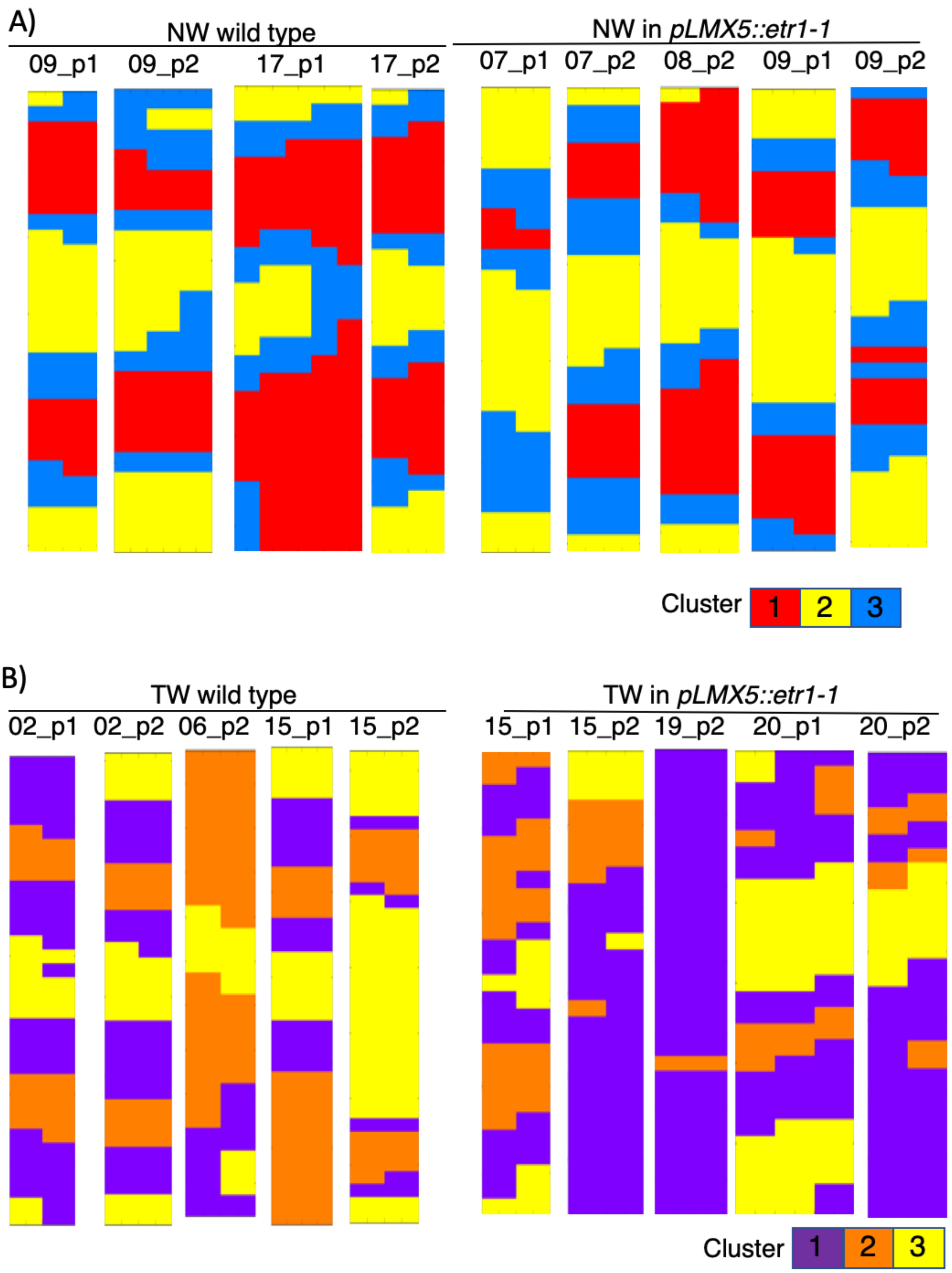

Supplementary Figure S2: k-means clustering based on four MCR-ALS resolved components (see Figure S1) and three clusters for normal wood (NW) and tension wood (TW) samples of wild type and ethylene insensitive *pLMX5::etr1-1* hybrid aspen trees. The clusters in A) correspond to: 1 (red): lignin and cellulose rich cluster (S-layer + middle lamella); 2 (yellow): lumen; 3 (blue): cellulose rich areas (S-layer). The clusters in B) correspond to: 1 (violet): cellulose rich areas (G-layer); 2 (orange): lignin and cellulose rich areas (S-layer + middle lamella); 3 (yellow): lumen. For the contribution of the MCR-ALS resolved components to the respective clusters, see component maps and centroid plots in Supplementary Figure S1. Numbers in the sample names correspond to the biological replicate tree, “p1” and “p2” indicate the two distinct positions scanned for every section. Pixel size is 1µm^2.

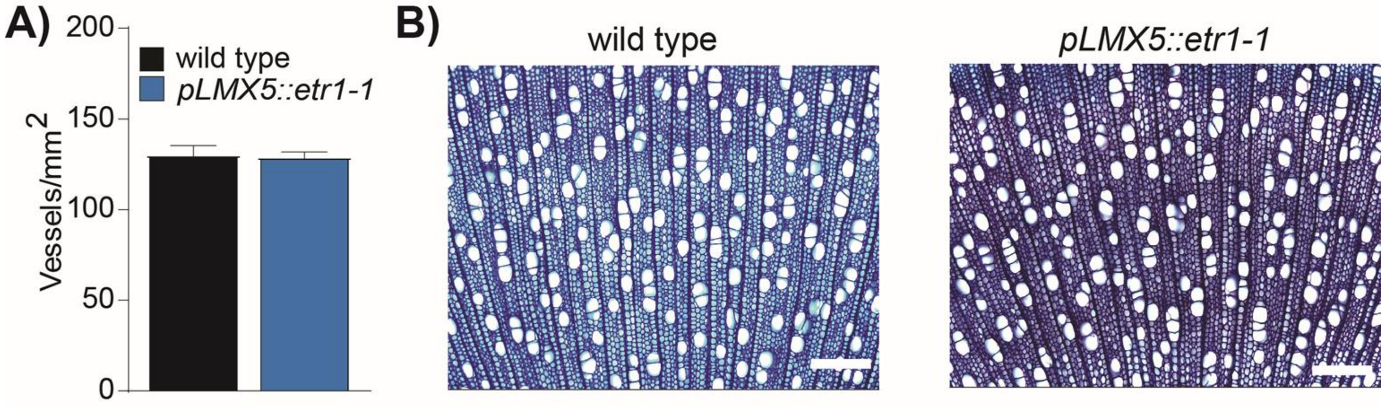

Supplementary Figure S3: Vessel density in normal wood of upright grown trees indicate no differences between *pLMX5::etr1-1* and wild type hybrid aspen trees. A) Average (±SD) vessel density in *pLMX5::etr1-1* and wild type trees. B) Representative 10X magnified images used for vessel density determination in each four trees per genotype. Scale = 50 μm

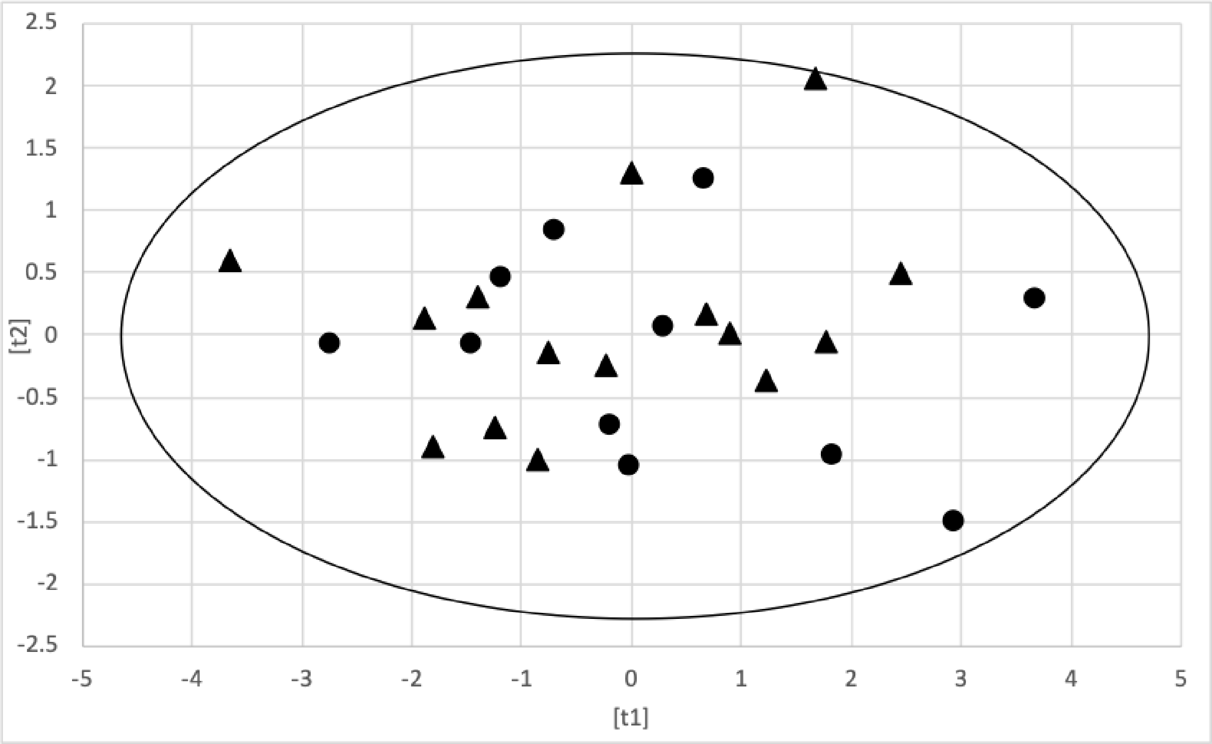

Supplementary Figure S4: First and second component of PCA of FT-IR single element detector data obtained from TW of wild type (triangles) and *pLMX5::etr1-1* (dots) hybrid aspen trees. No separation between the genotypes was observed. Model details: Autofit, 8 components, 26 observations. Principal Component 1 describes 70.4% of the variation, Component 2 describes 15.2% of the variation. R2X(cum)=0.995; Q2(cum)=0.981.

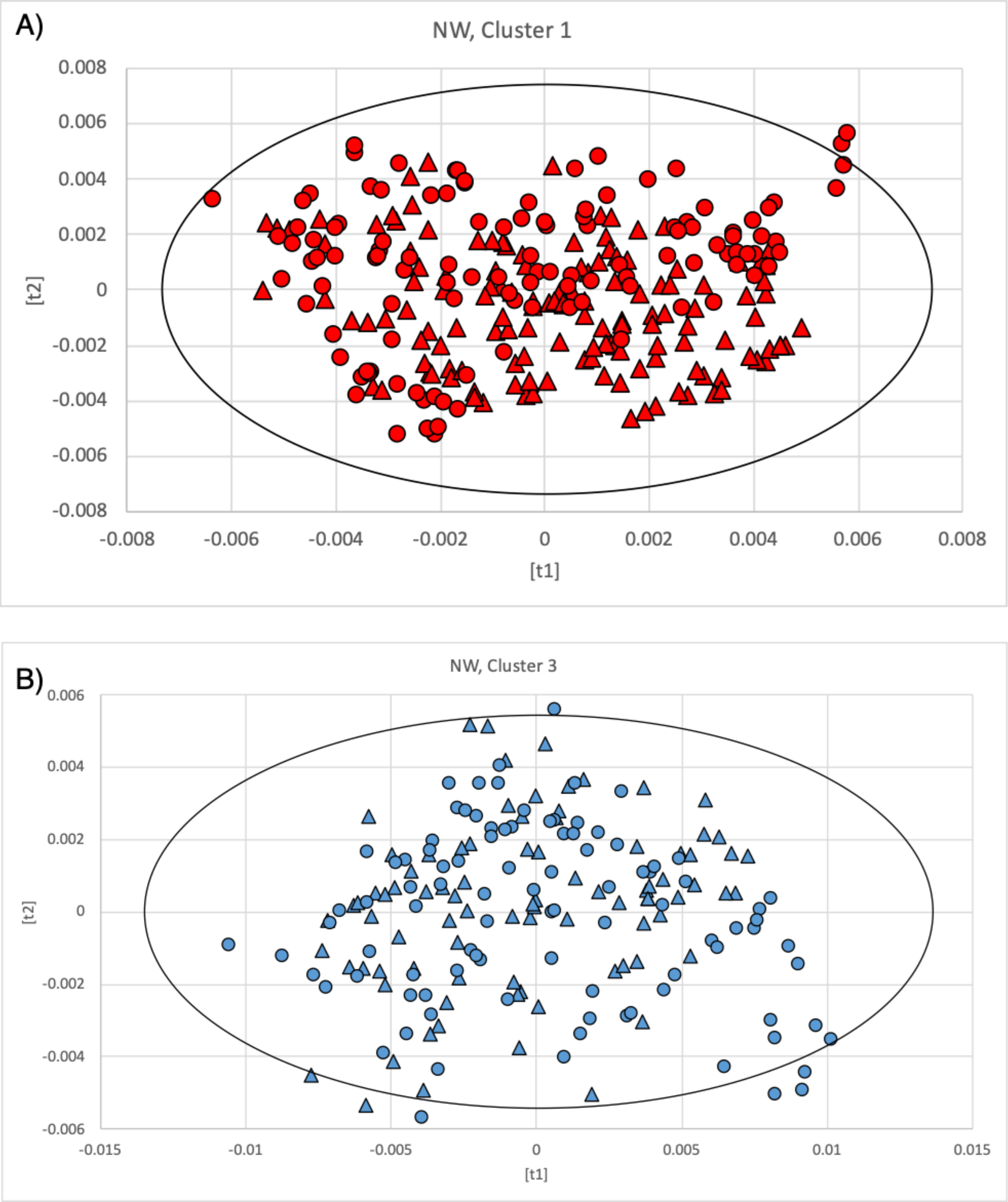

Supplementary Figure S5: Genotype comparison in NW by PCA for Cluster 1 (A, S-layer+Middle lamella) and Cluster 3 (B, S-layer) pixels for wild type (triangles) and *pLMX5::etr1-1* hybrid aspen trees (dots). There was no clear chemical difference in the NW cell wall layers between the two genotypes. Model details: A) Autofit, 43 components, 258 observations. Principal Component 1 describes 32.5% of the variation, Component 2 describes 25.4% of the variation. R2X(cum)=0.998; Q2(cum)=0.993.; B) Autofit, 43 components, 184 observations. Principal Component 1 describes 46.7% of the variation, Component 2 describes 12.8% of the variation. R2X(cum)=0.997; Q2(cum)=0.987.

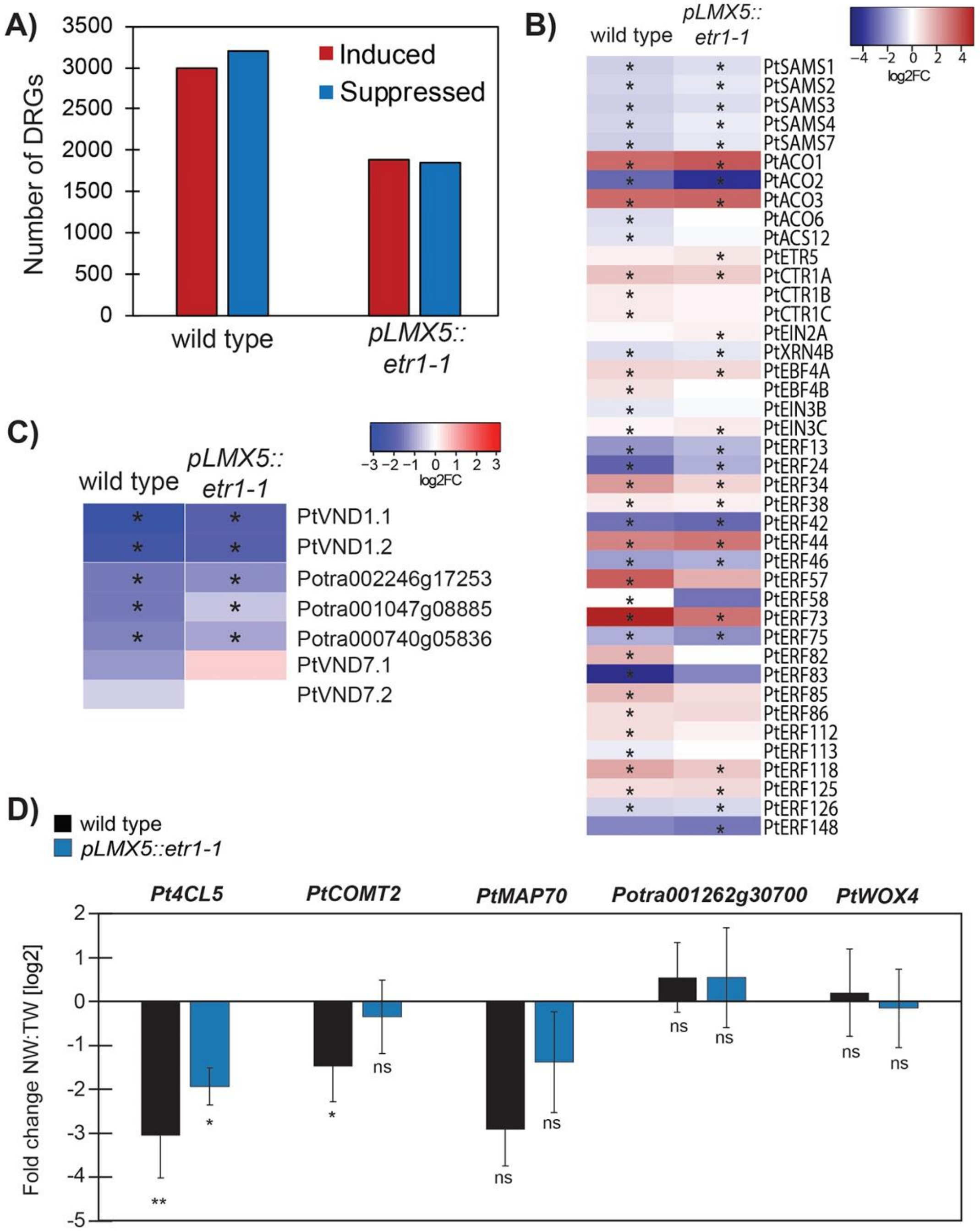

Supplementary Figure S6: Tension wood specific gene expression patterns in wild type and ETI trees. A) Number of up (red)- and down (blue)-regulated DRGs in tension compared to normal wood of wild type (WT) and the two ETI trees (Supplementary Table S1,S2). B) Expression change (log2FC) of VND family members in tension compared to normal wood (Supplementary Table S7). Asterisks indicate significant expression changes (pAdj<0.05 cutoffs).
